# Acute gastric dilatation with segmented abdominal paresis as a rare manifestation of herpes zoster: a case report and review of the literature

**DOI:** 10.1186/s13256-024-04487-2

**Published:** 2024-04-08

**Authors:** Toshihiko Yagyu, Yoshikazu Yakami, Tomoki Bando

**Affiliations:** Department of Gastroenterology, Higashi Osaka Hospital, 1-7-22, Chuo, Jyoto-Ku, Osaka, 536-0005 Japan

**Keywords:** Acute gastric dilatation, Gastroparesis, Herpes zoster, Segmental zoster paresis, Postherpetic pseudohernia

## Abstract

**Background:**

Herpes zoster is a common disease that can affect men and women at any age. Sensory neuropathy is the most common complication while motor neuropathy of the abdominal muscles is rare complication appearing in ~ 0.7% of patients. Furthermore, visceral nerve involvement causing gastroparesis is an extremely rare postherpetic complication. We present an extremely rare case of acute gastric dilatation with segmented abdominal paresis as a rare manifestation of herpes zoster infection.

**Case presentation:**

A 91-year-old Asian man was admitted to hospital with 2-day history of vomiting and left abdominal protrusion. He was previously treated for a rash on the left abdominal wall as herpes zoster infection with oral valaciclovir 2 weeks prior. On physical examination, characteristic herpes zoster rash scars and an ipsilateral abdominal bulge were observed on the left side. Computed tomography revealed no abdominal wall defect, mass, or stenosis. Remarkable distension of the stomach, asymmetrical left flank wall bulge, and a thinner abdominal wall on the left compared with the right side were shown. He was diagnosed as acute gastric dilatation owing to gastroparesis and segmental paresis of the abdominal musculature associated with herpes zoster infection. The patient showed significant improvement in symptoms and abdominal paresis within a month of conservative treatment, including nasogastric tube decompression and mosapride administration.

**Conclusion:**

Acute gastric dilatation with abdominal paresis is an extremely rare complication of herpes zoster infection, and to date there have been no reports in the literature. It alerts us that, when examining patients with abdominal bulge, we should be conscious of this rare pathology for the optical diagnosis, avoiding unnecessary invasive examination or surgical exploration.

## Background

While herpes zoster can occur at any age, complications such as acute gastric dilatation are particularly rare and underreported in elderly populations above 90 years, as seen in our case. Herpes zoster is induced by reactivation of varicella-zoster virus (VZV), which typically incubates in posterior roots of ganglia. It usually causes sensory symptoms and motor neuropathy may occur, although it is uncommon [1]. The incidence rate of abdominal paresis was ~ 0.7% [2], mimicking pseudohernia. Furthermore, visceral nerve involvement causing gastroparesis is an extremely rare postherpetic complication. There have been relatively few reports published so far [3–6]. We describe an exceptionally rare case of acute gastric dilatation (AGD) caused by gastroparesis with segmental zoster abdominal paresis. Since the prognoses of both neuropathy types are generally favorable with conservative therapy [7–9], early diagnosis and appropriate treatments are necessary. It is noted that attention must be paid to this rare pathology for the optical diagnosis to avoid unnecessary invasive examination or surgical exploration for examining patients with abdominal bulge. To the authors’ knowledge, this is the first case report in the literature. Additionally, we review the previous cases of postherpetic gastroparesis. Written informed consent was obtained from the patient for publication of this case report and any accompanying images. A copy of the written consent is available for review by the Editor-in-Chief of this journal. The case report is exempt from ethical approval in our institution.

## Case presentation

**Fig. 1:**
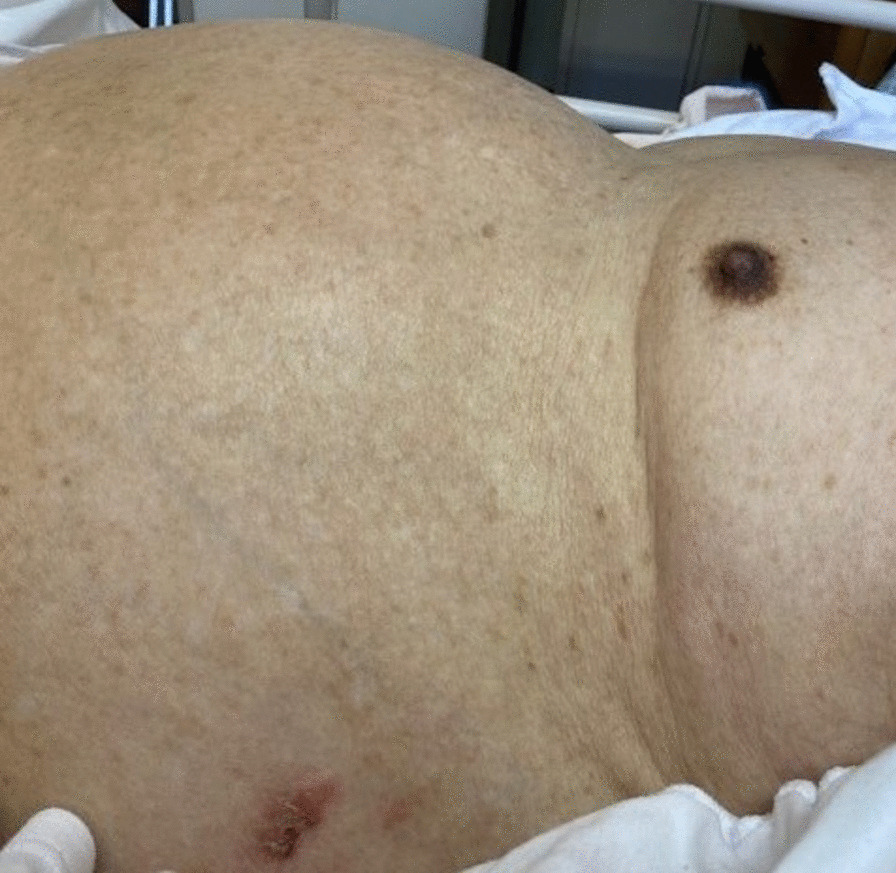
Herpes zoster skin eruption in the 10th left thoracic dermatome on admission

**Fig. 2:**
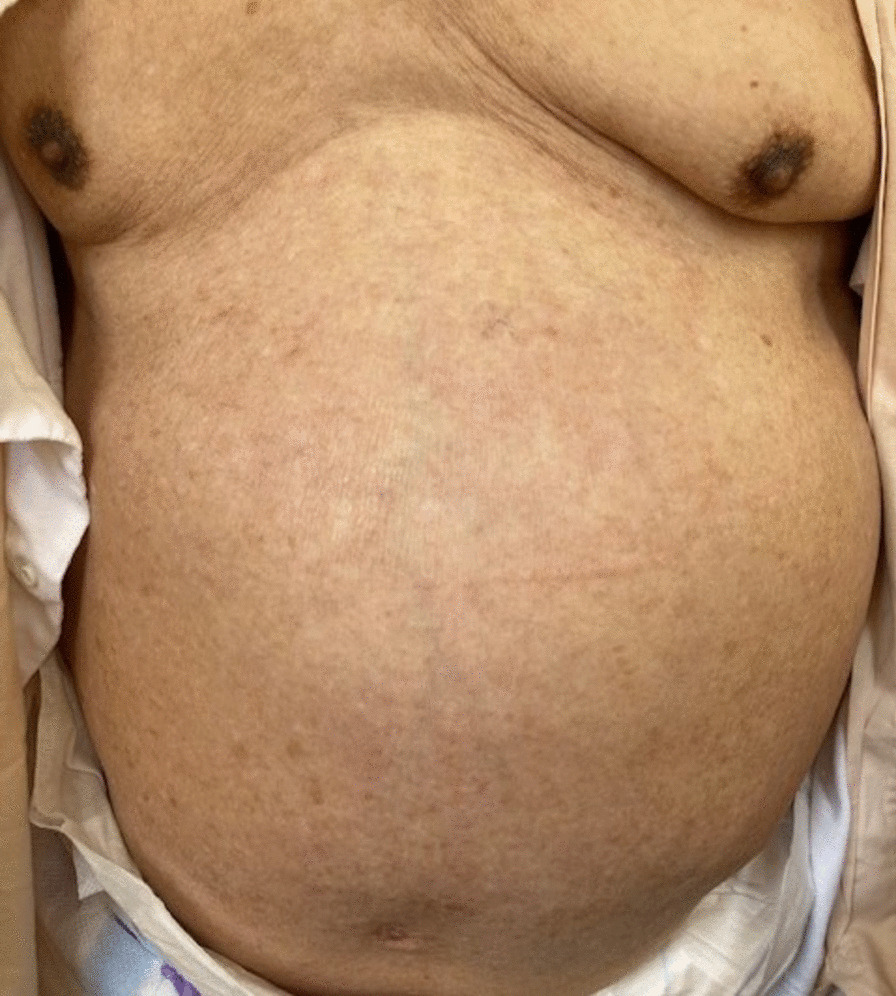
Prominent bulging on the left flank

**Fig. 3:**
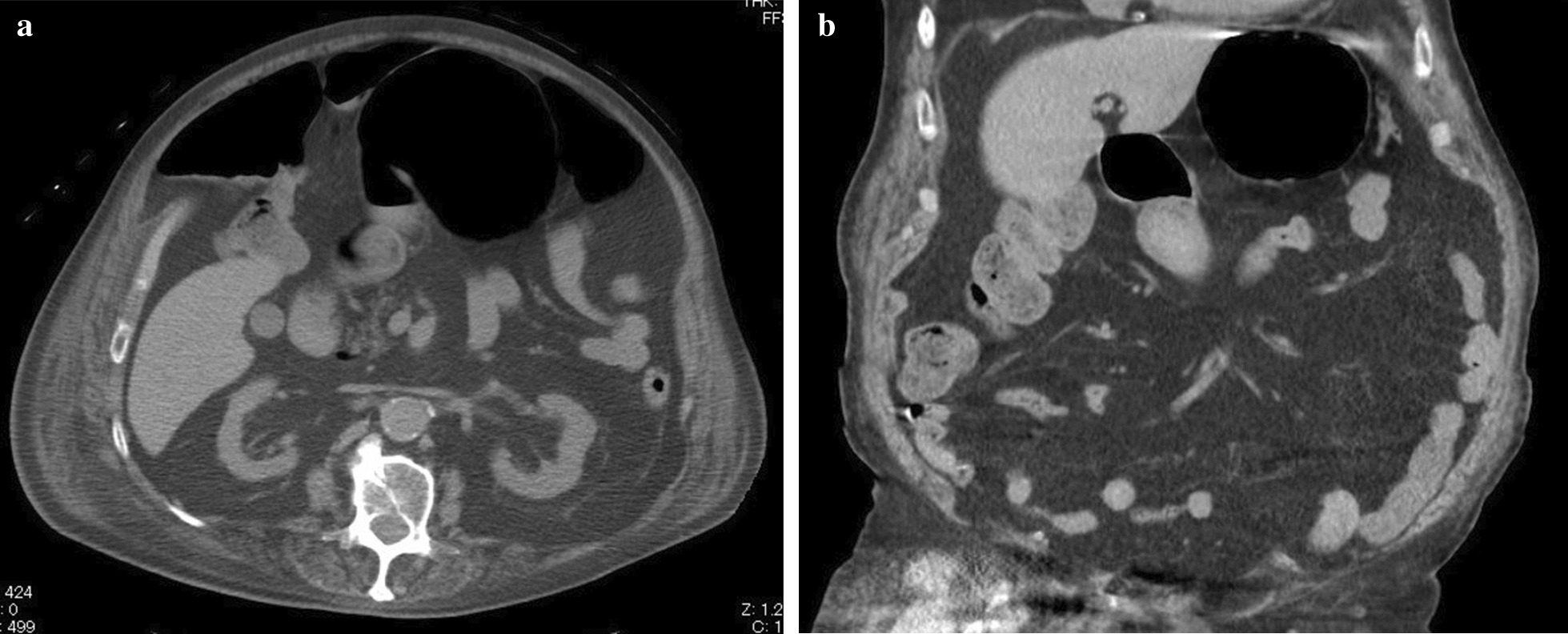
Plain abdominal CT revealing distended stomach and thinning of the left abdominal wall muscles. **a **Axial view; **b** Coronal view

A 91-year-old Asian man with a history of a hypertension was admitted to hospital with 2-day history of vomiting and protrusion in the left abdominal wall. He had a rash in his left abdominal wall, was diagnosed with herpes zoster infection 2 weeks before admission, and was treated with oral valaciclovir (1000 mg three times daily for 7 days). He had no history of diabetes mellitus, psychogenic disorders, or abdominal surgery. He denied any prior symptoms of delayed gastric emptying, such as postprandial fullness or early satiety. His heart rate, respiratory rate, blood pressure, and temperature were normal. Physical examination revealed a healed herpetic skin rash on the left side of the abdominal wall in the area innervated by the 10th thoracic nerves (Fig. 1). The ipsilateral abdominal wall was remarkably flaccid and distended, with the disappearance of abdominal wall reflexes (Fig. 2). Bowel sounds were decreased and hematologic and biochemical tests were unremarkable, except for elevation of serum anti-VZV antibody titer. Serum thyroid hormone was within normal levels. Computed tomography (CT) was performed to rule out structural causes of the abdominal protrusion and endoscopy was used to confirm gastric distension and rule out mechanical obstruction. CT revealed no abdominal wall defect, mass, or stenosis, although remarkable distension of the stomach without volvulus was confirmed. The left flank wall bulged asymmetrically and the abdominal muscle was thinner than that on the right side (Fig. 3). Upper endoscopy showed esophagitis, gastritis, and massive gastric enlargement with no mechanical obstruction. A biopsy of the stomach was consistent with nonspecific active inflammation without inclusion bodies or giant cells. Colonoscopy excluded any obstructive process. Although nuclear scintigraphy for quantifying gastric emptying was not available, solid meal residue was still recognized in gastroscopy 1 week after admission. Structural causes of the abdominal protrusion were ruled out with CT, and endoscopy was used to confirm gastric distension and rule out mechanical obstruction. Given the patient’s age, lack of abdominal surgery history, and recent herpes zoster infection, these symptoms led us to consider a neuropathic origin for his gastric and abdominal symptoms. He was diagnosed as AGD owing to gastroparesis and segmental paresis of the abdominal musculature, both of which were associated with herpes zoster. Considering the patient’s age, absence of mechanical obstruction, and the neuropathic nature of his condition, we opted for conservative management to avoid the risks associated with surgical intervention. At 3 days after nasogastric tube (NGT) decompression and 2 weeks after parenteral nutrition, he tolerated feeds without vomiting. Gastric distension and ipsilateral abdominal bulge were alleviated 1 month after conservative treatment with the administration of mosapride (5 mg three times daily). He was discharged 41 days after admission with uneventful recovery.

## Discussion

Gastroparesis is an extremely rare manifestation of postherpetic neurological complication. There have been relatively few reports published so far. We searched PubMed using the following terms in different combinations: “postherpetic,” “gastroparesis,” “segmental zoster paresis,” “varicella zoster,” and “Herpes Zoster.” Clinical trials, reviews, and case reports were collected and reviewed to reveal this unusual clinical entity. Only articles in English were included. In total, four articles that described four cases were identified [3–6]. No abdominal paresis accompanied them. Unlike previously reported cases where gastroparesis did not accompany abdominal paresis, our case uniquely presents both complications simultaneously in a nonagenarian patient. Including our case, the age of the patients ranged from 50 to 91 years old with an average age of 70.8 years, and the male to female ratio was 5:0. Gastroparesis occurred 4 days to 3 weeks after the appearance of skin rash, except in one simultaneous case. Urinary retention was accompanied in one case. Regular antiviral therapy was administered in three cases. Metoclopramide was administered in four cases and its efficacy was shown with a radionuclide gastric emptying test [4] though it was not applicable for poor renal function in our case. The prognosis of gastroparesis was generally favorable [8]. All cases showed improvement in their symptoms with conservative treatment within 1–3 months. In one case, percutaneous endoscopic gastrostomy was performed before the diagnosis of gastroparesis. To the authors’ knowledge, a case of acute gastric dilatation with abdominal paresis as a post herpetic neurological complication has not been published in literature, and therefore our case could be the first case report. Herpes zoster usually involves sensory neurons causing pain and paresthesia and visceral neuropathies are uncommon, while on the other hand, urinary retention or constipation with concomitant intestinal pseudo-obstruction have been reported [1, 7, 9, 10]. The site of the neurologic lesion causing the gut manifestations is uncertain and the pathologies of motor and visceral neuropathy is still controversial [10]. However, there are hypothesizes for the viral transmission to the nerves, such as direct viral spreading, vessel vasculitis, shredding from oral mucus membrane, dissemination, and transaxonal extension [11–15]. According to Li *et al*. [16], the pathophysiology may involve viral spread along nerve fibers, which is consistent with the observations in our case. As for treatment of gastroparesis, prokinetic medications are mainstays. Although dopamine-2 antagonists and 5-HT4 receptor agonists were considered appropriate therapies, there have been only few large scale trials of prokinetic drugs in gastroparesis [8, 17–19]. Physicians should be reminded about the side effects and risks prior to prescription. In the present case, we used mosapride as a prokinetic drug considering the patient age and renal function, though it was difficult to verify its efficacy.

AGD, first reported by Duplay in 1833 [20], is a rare but serious condition with high mortality owing to development of sequelae, such as gastric emphysema, gangrene, and perforation [20–22]. Delay in operative management has resulted in mortality that was as high as 80% in published data [23]. Etiologies have been postulated, including eating disorders, postoperative gastric distension, anesthesia, debilitation, mechanical obstruction, and gastroparesis [22, 24–26]. This condition, without intervention, results in high mortality from gastric ischemia. Immediate decompression via NGT is the first priority procedure for AGD for the prevention of ischemia, which leads to perforation [20, 27]. In addition, upper endoscopy, with perforation excluded, should be feasible in evaluating the etiology of dilatation, assessing the ischemic change of mucosa and decompressing the stomach [20, 22]. Relieving of distension and management of gastric necrosis or perforation are mainstays of AGD treatment. It is notable to monitor gastric necrosis even after relieving distension.

This case, accompanied with segmental abdominal paresis mimicking an abdominal hernia, raised diagnostic and treatment challenges that were experienced by the emergency team and radiologist. Cautious confirmation of medical history would be emphasized and appropriate management should be instigated on the basis of basic surgical principles.

## Conclusion

This case underlines the importance of considering neuropathic complications in elderly patients with herpes zoster, even when presenting with atypical abdominal symptoms. Presentation of this pathology would be notable for delayed onset of manifestation after herpes zoster infection. Understanding on the neurologic complications secondary to herpes zoster should be emphasized to avoid unnecessary invasive investigations and surgery, and it would lead to optimal treatments with favorable prognosis.

## Data Availability

There are no additional data available for this study.

## Abbreviations

VZV: Varicella-zoster virus
AGD: Acute gastric dilatation
CT: Computed tomography
NGT: Nasogastric tube
